# The Role of Nuclear Matrix Proteins Binding to Matrix Attachment Regions (MARs) in Prostate Cancer Cell Differentiation

**DOI:** 10.1371/journal.pone.0040617

**Published:** 2012-07-11

**Authors:** Paola Barboro, Erica Repaci, Cristina D’Arrigo, Cecilia Balbi

**Affiliations:** 1 IRCCS Azienda Ospedaliera Universitaria San Martino IST-Istituto Nazionale per la Ricerca sul Cancro, Department of Diagnostic Technologies, Genoa, Italy; 2 C.N.R., Istituto per lo Studio delle Macromolecole, ISMAC, Sezione di Genova, Genoa, Italy; University of Nebraska Medical Center, United States of America

## Abstract

In tumor progression definite alterations in nuclear matrix (NM) protein composition as well as in chromatin structure occur. The NM interacts with chromatin via specialized DNA sequences called matrix attachment regions (MARs). In the present study, using a proteomic approach along with a two-dimensional Southwestern assay and confocal laser microscopy, we show that the differentiation of stabilized human prostate carcinoma cells is marked out by modifications both NM protein composition and bond between NM proteins and MARs. Well-differentiated androgen-responsive and slowly growing LNCaP cells are characterized by a less complex pattern and by a major number of proteins binding MAR sequences in comparison to 22Rv1 cells expressing androgen receptor but androgen-independent. Finally, in the poorly differentiated and strongly aggressive androgen-independent PC3 cells the complexity of NM pattern further increases and a minor number of proteins bind the MARs. Furthermore, in this cell line with respect to LNCaP cells, these changes are synchronous with modifications in both the nuclear distribution of the MAR sequences and in the average loop dimensions that significantly increase. Although the expression of many NM proteins changes during dedifferentiation, only a very limited group of MAR-binding proteins seem to play a key role in this process. Variations in the expression of poly (ADP-ribose) polymerase (PARP) and special AT-rich sequence-binding protein-1 (SATB1) along with an increase in the phosphorylation of lamin B represent changes that might trigger passage towards a more aggressive phenotype. These results suggest that elucidating the MAR-binding proteins that are involved in the differentiation of prostate cancer cells could be an important tool to improve our understanding of this carcinogenesis process, and they could also be novel targets for prostate cancer therapy.

## Introduction

Abnormal nuclear organization and alterations in the amount and distribution of heterochromatin have long been recognized as hallmarks of human cancer [1]; however, at present we do not know the exact causes of these modifications, nor do we know how the activity/silencing of thousands of genes is orchestrated. In eukaryotes, the genome is compartmentalized into chromatin domains by the attachment of chromatin to a supporting structure: the nuclear matrix (NM). The interactions between chromatin and the NM occur via AT-rich DNA sequences called matrix attachment regions (MARs). The MARs function in several processes including organizing chromatin loops, augmenting gene expression and facilitating replication [2]. Not all potential MARs are bound to the NM or participate in the organization of loop attachment regions. MAR binding is a dynamic event that is cell type and/or cell cycle-dependent and can allow the regulation of distant genes in a coordinated manner [3]. Several MAR-binding proteins have been identified, some of which are dramatically deregulated in tumor cells. Often their expression is also significantly correlated with aggressive tumor phenotypes. Likewise, modifications in the interactions between NM proteins and MARs might be related to the large-scale chromatin reorganization observed during carcinogenesis. This has prompted a rising interest in MARs and MAR-binding proteins as potential targets for antineoplastic drugs [2].

Recently, we have demonstrated that in the early stages of rat liver carcinogenesis, large-scale chromatin reorganization is related to morphological and protein composition alterations of the NM. These changes modify the ability of NM proteins to bind RNA and DNA-containing MAR sequences [4]. Moreover, these alterations are synchronous with changes in the organization of lamins in the nucleoplasm. In normal hepatocytes, the lamins are assembled into filaments that form an orthogonal lattice, whereas in transformed hepatocytes the two-dimensional local order is lost [5].

Prostate carcinoma (PCa) represents a major health concern because its incidence continues to increase, and there are no biomarkers currently able to distinguish indolent tumors from aggressive ones. Androgen ablation is the most common therapeutic approach to PCa. Unfortunately, after a few years of treatment, the disease progresses in most patients who then acquire an androgen-independent phenotype for which there are no treatments available [6]. An understanding of the pathways that lead to androgen independence is therefore critical to developing new therapies. Work carried out in our laboratory and others to search for PCa markers with improved diagnostic and prognostic features has identified several NM proteins that are differentially expressed in PCa with respect to non-tumor tissue; moreover, a few proteins were significantly correlated with tumor aggressiveness and/or risk of biochemical progression [7], [8].

In this study, we used a proteomic approach together with two-dimensional Southwestern blotting (SWB) and confocal analyses to characterize the bond between NM proteins and MARs in three human PCa cell lines representing models of different stages of PCa progression: the well-differentiated androgen-responsive LNCaP cell line, the intermediate-differentiate 22Rv1 cells expressing androgen receptor (AR) but androgen-independent and finally the poorly differentiated and strongly aggressive PC3 which does not express AR. These cell lines are a good model system to study PCa progression as more than 70% of the NM proteins expressed match those isolated from PCa tissues [9]. Here we provide evidence that there is an inverse relationship between complexity of NM protein composition and the grade of differentiation of cell line and that the NM interactions with MAR sequences change during differentiation, which modifies chromatin loop dimensions and consequently gene expression.

**Table 1:** Specificity of the antibodies used in this study for WB and SWB analysis.

Antigen	Type	Label	Host	Clone	Company	Dilution
hnRNP I	Polyclonal		Goat	N-20	Santa Cruz	1∶400
hnRNP K	Monoclonal		Mouse	D-6	Santa Cruz	1∶4000
hnRNP M	Monoclonal		Mouse	1D8	Santa Cruz	1∶500
hnRNP U	Monoclonal		Mouse	3G6	Santa Cruz	1∶800
Lamin A/C	Polyclonal		Goat	N-18	Santa Cruz	1∶600
Lamin B	Polyclonal		Goat	C-20	Santa Cruz	1∶1500
PARP1/2	Polyclonal		Rabbit	H-250	Santa Cruz	1∶300
SATB1	Polyclonal		Goat	E-15	Santa Cruz	1∶200
Matrin 3	Polyclonal		Rabbit		Bethyl Laboratories	1∶1000
Goat Ig G	Polyclonal	Peroxidase	Donkey		Santa Cruz	1∶1000
Mouse Ig G	Polyclonal	Peroxidase	Goat		Santa Cruz	1∶1000
Rabbit Ig G	Polyclonal	Peroxidase	Swine		DAKO	1∶1000

## Materials and Methods

### Cell Culture

LNCaP, 22Rv1 and PC3 prostate carcinoma cell lines were obtained from ATCC (CRL-1740, CRL-2505 and CRL-1435, respectively; Rockville, MD, USA) and maintained in RPMI-1640 without phenol red (Celbio, Milan, Italy) containing heat-inactivated 10% fetal bovine serum (charcoal stripped), 1% penicillin, 1% streptomycin and 1% glutamine. LNCaP and 22Rv1 medium was also supplemented with 10 mM HEPES, 1 mM sodium pyruvate and 4.5 mg/ml glucose. The passage numbers at which LNCaP cells were placed on maintenance medium ranged from 24 to 34. Cells were cultured in a monolayer in the presence of 0.1 nM 5-α-dihydrotestosterone (SIGMA, St. Louis, MO, USA) at 37°C in 5% CO_2_. All experiments were carried out using cells from an exponential phase culture.

### Isolation of the NM

For one- or two-dimensional polyacrylamide gel electrophoresis (1D or 2D-PAGE), the NM was isolated according to Barboro *et al*. [10]. Protein concentrations were determined using the Bio-Rad (München, Germany) protein microassay with bovine serum albumin as a standard. For confocal microscopy, the NM was extracted *in situ* as described by Zeng *et al*. [11].

**Figure 1:**
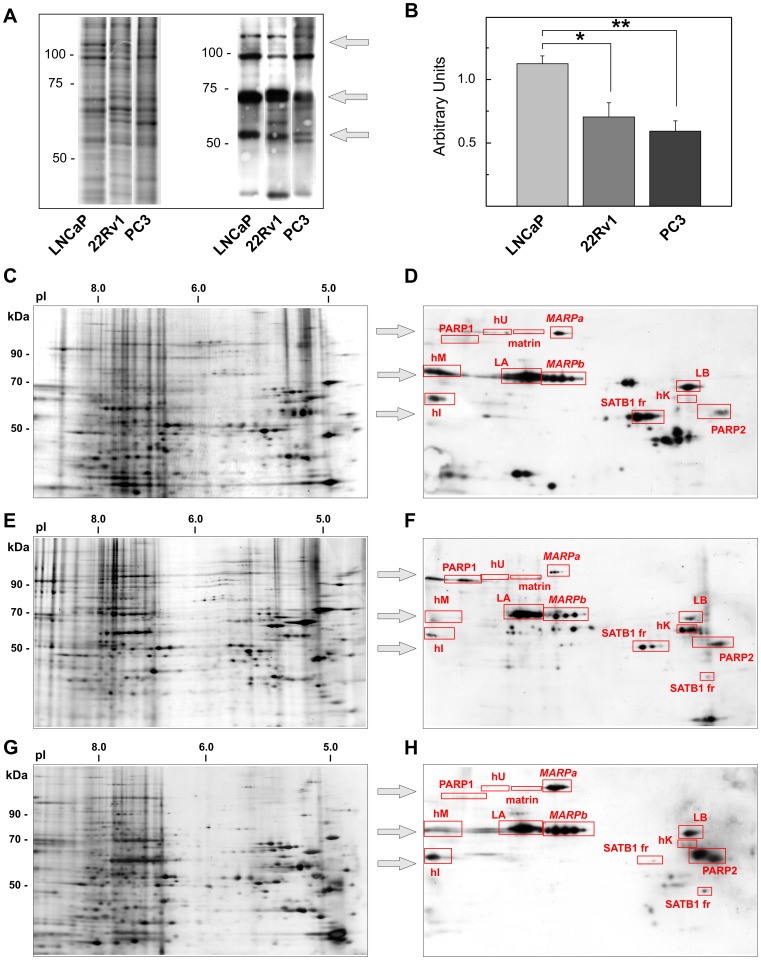
NM proteins binding the XmnI sequence in PCa cell lines. (A) Representative Deep Purple-stained 1D gel and the corresponding SWB. The arrows on the right indicate the three principal bands arising in 1D SWB, each of which corresponds to several spots in 2D as evident in (D), (F) and (H). (B) The comparison between the relative quantity of XmnI binding to NM proteins in the different cell lines. Ordinate represents the mean±SE of the relative amounts of XmnI as determined by quantitative analysis of three different preparations. The decrease in 22Rv1 and PC3 with respect LNCaP cells was significant (*P = 0.004, **P<10^−5^). (C, E and G) Representative 2D silver-stained gel maps and (D, F and H) SWB of NM proteins extracted from LNCaP (C, D), 22Rv1 (E, F) and PC3 (G, H) cells. The proteins identified are highlighted in red boxes. The three arrows show the three groups of proteins pointed out in (A). L, lamin; h, hnRNP, fr, fragments.

### Preparation of a DNA Probe

A highly repetitive DNA sequence of 370 bp (XmnI) obtained from the S/MAR transaction DataBase [http://smartdb.bioinf.med.uni-goettingen.de] release 2.3 (accession number SM0000134) was used as a probe for DNA binding experiments. XmnI is an AT-rich DNA sequence within a base-unpairing region (BUR) and is able to bind the same proteins that bind the MAR-containing DNA co-isolated with the NM, as we have previously demonstrated [4]. Plasmid pUC57 with the XmnI sequence was constructed by GenScript Corporation (Piscatway, NJ, USA). *Escherichia coli* TOP F10’ was transformed with pUC57, and transformed cells were selected on agar plates supplemented with ampicillin. Plasmids were isolated using the Qiagen Plasmid Maxi kit, restriction-digested with *Xmn*I and subsequently gel-purified from agarose using a High Pure PCR product purification kit (Roche Diagnostics, Mannheim, Germany).

DNA samples were biotinylated by the nick translation procedure (BioNick DNA Labeling System, Invitrogen/LifeTechnologies, San Diego, CA). Unincorporated nucleotides were removed from the sample using a Sephadex G-25 spin column (Roche Diagnostics).

### Nuclear Halo Preparation and Halo-fluorescence in Situ Hybridization (FISH) Technique

Nuclear halos were prepared according to the method of de Belle *et al*. [12] with minor modifications. Briefly, LNCaP and PC3 cells were washed two times in phosphate buffered saline (PBS) and the nuclei were extracted by incubating the cells in 100 mM KCl, 300 mM sucrose, 10 mM PIPES, pH 6.8, 3 mM MgCl_2_, 0.5% Triton X-100 at 4*°*C for 20 min. Then, 3×10^5^ nuclei were cytospun onto slides for 10 min at 100×*g.* The slides were immersed for 4 min in a solution containing 2 M NaCl, 10 mM PIPES, pH 6.8 and 10 mM EDTA and then rinsed for 2 min by a series of 10X, 5X, 2X and 1X PBS, pH 7.4. After fixation with 3.7% formaldehyde in PBS, the DNA was stained with DAPI and the relative halo size of each nucleus was determined as reported by Guillou *et al*. [13].

**Figure 2:**
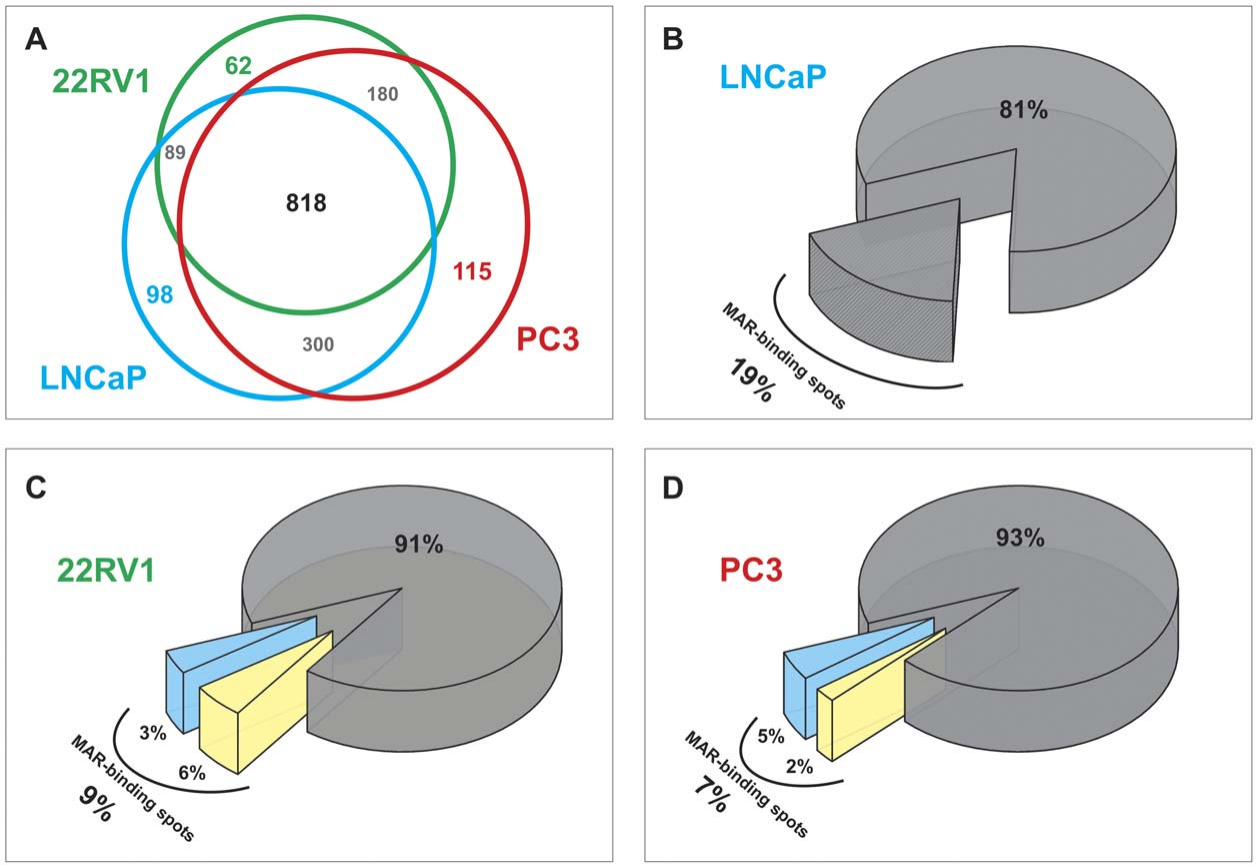
Analysis of the differentially expressed NM proteins in LNCaP, 22Rv1 and PC3 cells. (A) Venn diagram showing the number of protein spots visualized in each cell line. Numbers in the overlapping regions represent common spots. (B–D) Pie charts showing the percentage of spots that bind the XmnI sequence in three cell lines. The colors cyan and yellow, denote the spots that were, with respect LNCaP cells, differently expressed or with no difference, in 22Rv1 (C) or PC3 (D), respectively.

To examine the distribution of the XmnI sequences into nucleoids, the halo-FISH technique was applied. After halo extraction and fixation, the slides were incubated with 70% formamide, 2x saline-sodium citrate (SSC) and 50 mM sodium phosphate, pH 7.0 at 73°C for 3 min followed by 50% formamide, 2x SSC and 50 mM sodium phosphate, pH 7.0, at 73°C for 1 min. The hybridization mixture (4 ng/µl biotinylated XmnI probe, 2x SSC, 1 mg/ml competitor DNA, 10% dextran sulfate and 25% formamide) was heat denatured separately for 5 min at 73°C, rapidly cooled on ice and applied to the specimen. After an overnight incubation at 37°C, the slides were washed three times with 50% formamide and 2x SSC, pH 7.0, at 42°C for 5 min followed by another three washings with 0.1x SSC at 60°C for 5 min. FISH detection was carried out with streptavidin conjugated with CF^TM^555 (1∶100 dilution, Biotium, Hayward, CA), while total DNA was counterstained with DAPI. The samples were analyzed by light microscopy using a Leica DM LB2 epifluorescence microscope (Wetzlar, Germany) equipped with a 40x objective. Images were captured with an Olympus DP70 camera and processed with WCIF ImageJ v1.43 software (http://www.uhnresearch.ca/facilities/wcif/imagej/).

### Gel Electrophoresis

1D and 2D-PAGE of the NM proteins were carried out as previously described [14]. The gels were either stained for protein pattern analysis or processed for Western blot (WB) or SWB. The lamin B phosphorylation pattern was performed by a multiplexed proteomic analysis. The gels were initially incubated with Pro-Q Diamond (Molecular Probe Inc., Eugene, OR, USA), a phosphoprotein-specific dye, followed by incubation with SYPRO Ruby dye (Molecular Probe Inc.) for total protein visualization, as recommended by the manufacturer.

### SWB and WB Procedures

SWB was performed as previously reported [4]. Briefly, a 1D or 2D-PAGE gel was electrotransferred to a Hybond-P membrane (GE Healthcare, Piscataway, NJ). The membrane was incubated in buffer A (150 mM NaCl, 10 mM Tris-HCl, pH 7.5, 10 mM MgCl_2_ and 0.5% Tween-20) for 2 h at room temperature and then incubated overnight at 37°C in the same buffer containing 50 ng/ml of DNA probe and a 500-fold excess of a non-specific competitor (sonicated herring sperm DNA). The membrane was then washed with three changes of buffer A over a period of 60 min. Before detection, the membranes probed with biotinylated DNA were incubated for 30 min with horseradish peroxidase-linked streptavidin (Invitrogen, Carlsbad, CA) diluted 1∶1200 in buffer A and were then washed four times with the same buffer for 15 min at room temperature. Non-radioactive detection of DNA bound to the NM was carried out with the enhanced ECL Plus Western Blotting Reagents and revealed using Hyperfilm ECL (GE Healthcare) with the same exposure conditions.

**Figure 3:**
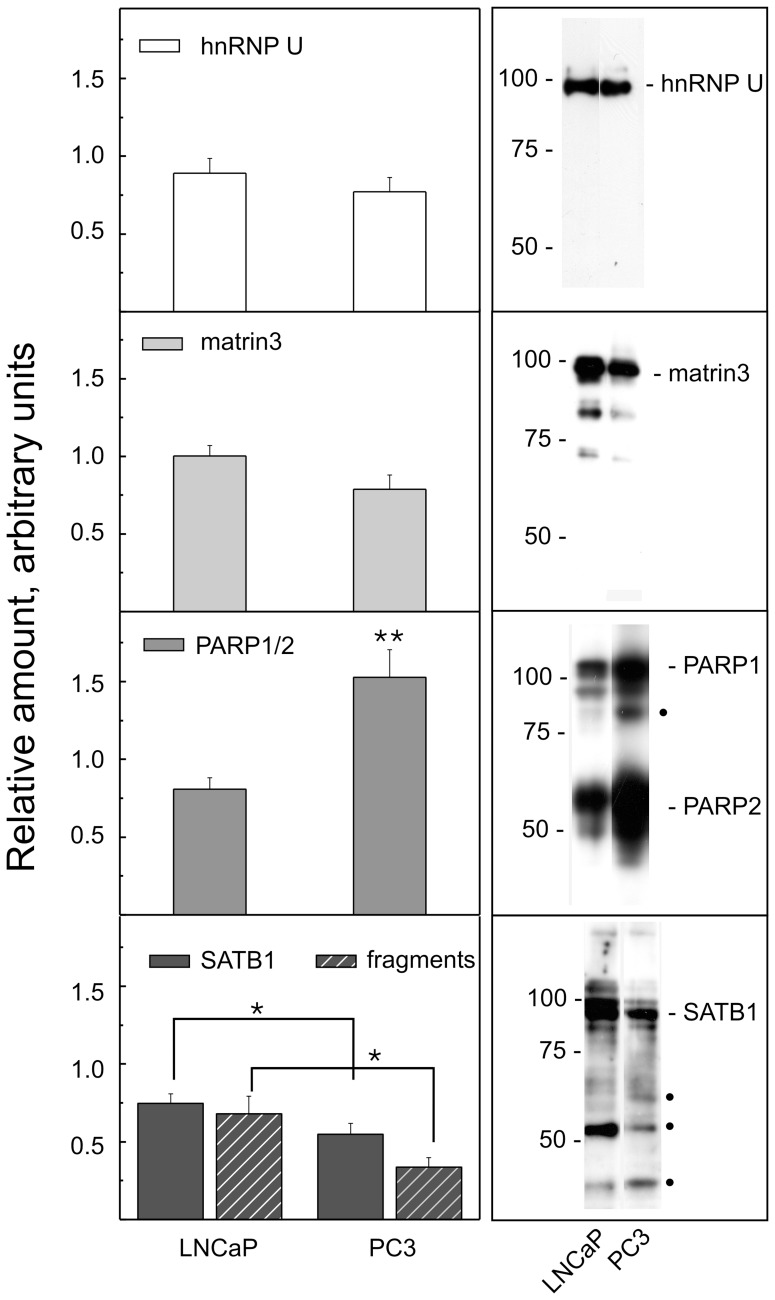
Expression levels of hnRNP U, Matrin3, PARP and SATB1 in LNCaP and PC3 cells. The ordinates represent the mean±SE of the relative amounts of these proteins as determined by quantitative analysis of three to six WBs carried out utilizing at least three different preparation of NM (*P≤0.05; **P<0.0005). Representative WBs are shown on the right; the major proteolytic fragments of PARP1 and SATB1 are marked by full dots. The relative molecular weights of standard proteins in kDa are reported.

For WB, proteins separated by 1D or 2D-PAGE were transferred to a Hybond-P membrane (GE Healthcare), and immunodetection was carried out using the antibodies reported in Table 1. Immunoreactive spots or bands were detected using ECL. After 1D WB analysis, the relative amount of each protein under study was obtained by normalizing the integrated optical density by ECL with the integrated optical density of the total amount of NM proteins determined by the Deep Purple staining gel as previously described [4]. Briefly, equal quantities (8 µg) of the same preparation were loaded on two 1D-PAGE gels and submitted to electrophoresis. One gel was stained with Deep Purple and densitometric scans were performed in a Molecular Imager FX scanner (Bio-Rad) and the total amounts of protein (*A*) were evaluated by integration of the optical density curve. The second gel was blotted and immunoreactive bands were detected using Hyperfilm ECL films (GE Healthcare), which exhibit a linear response to light produced from enhanced chemiluminescence. The relative amounts of proteins understudy were obtained by normalizing the integrated optical density by ECL (*B*) to the integrated optical density (*A*) of the corresponding Deep Purple-stained gel. This method allowed us to obtain quantitative results. The comparison of the relative amounts of the proteins was performed by exporting the single values of each film and analyzing them using Student’s t-test within the OriginPro 7.5 software.

### Image Analysis of 2D Gel Spot Patterns

All of the silver-stained 2D gels were digitized with a GS-800 densitometer (Bio-Rad) using the same scanning conditions. Spot detection, gel alignment and normalization were carried out using the software package PDQuest (Ver. 7.3.0, BioRad). Differential analysis was performed by grouping 2D gels into three classes, each containing four to six gels obtained from four to five different NM preparations isolated from LNCaP or 22Rv1 or PC3 cells. Reproducibility of the gels, expressed as a mean percentage of matched spots within each class ± SD, was 87±5% for LNCaP, 83±3% for 22Rv1 and 83±1% for PC3. The Mann-Whitney test was used to detect over- or under-expressed spots; P<0.05 was considered statistically significant and only protein spots whose expression was 2-fold or more deregulated were analyzed. To compensate for subtle differences in sample loading and gel staining, the volume of each spot was normalized according to total quantity in valid spots in each gel.

The PDQuest analysis software was also used to detect proteins that bound antibodies in 2D WB by matching antigen spots in autoradiographs with protein spots in replica 2D SWB.

**Figure 4:**
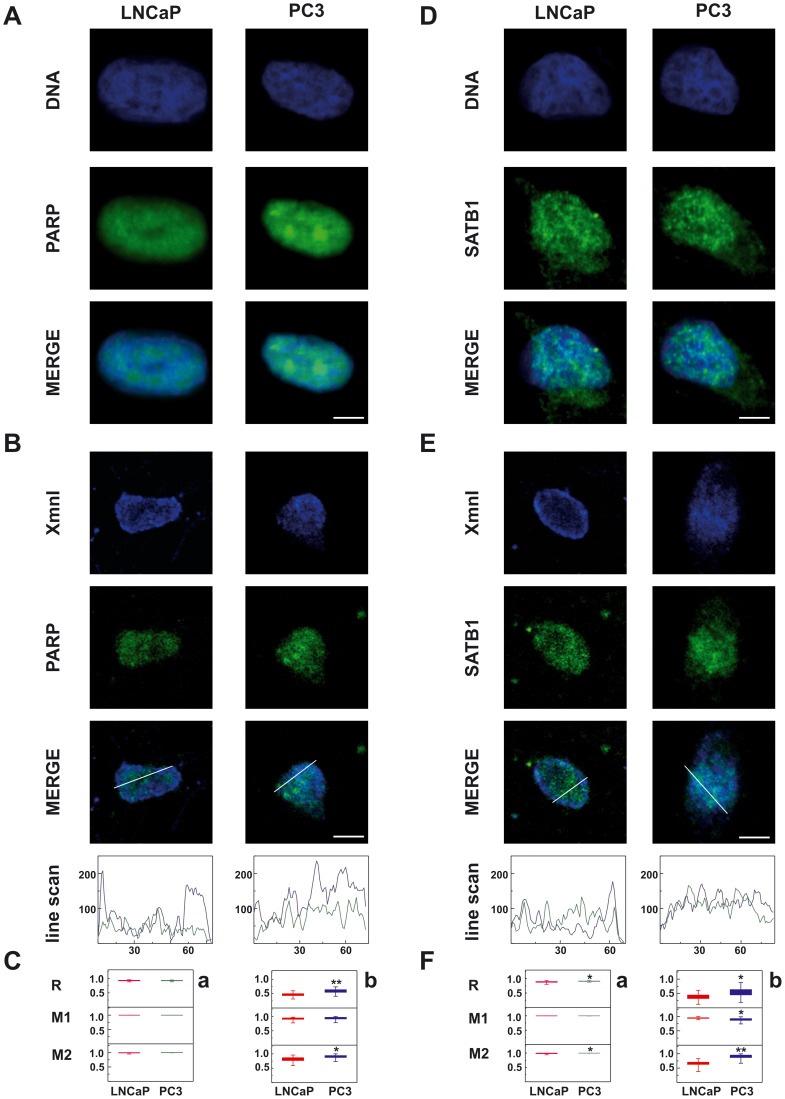
Spatial distribution of PARP and SATB1 in relation to DNA or XmnIsequence. (A, D) Whole cells stained by dual-color immunofluorescence. (B, E) NM prepared *in situ* and stained by immuno-FISH. (A, B) Confocal microscope analysis of the localization of PARP (green) and DNA or XmnI sequence (blue). (D, E) Localization of SATB1 (green) and DNA or XmnI sequence (blue). In the bottom of panels B and E the intensity profile line scans, performed between the white crosses of the NM as indicated on confocal merge images in B and E, are shown. The ordinate represents the fluorescence intensity in arbitrary units, the abscissa represents the distance in pixels. The bars correspond to 5 µm. (C, F) Scatter plots showing quantification analyses of the colocalization of PARP/DNA (a), PARP/XmnI (b), SATB1/DNA (a), or SATB1/XmnI (b), respectively. R corresponds to Pearson’s correlation coefficient; M1 to the fraction of protein being studied overlapping the DNA or XmnI and M2 the fraction of DNA or XmnI overlapping the protein. Horizontal lines show the mean values±SE of 20 fields (122–226 total NMs) replicated in two different experiments (*P≤0.03, **P<0.001).

### Confocal Laser Scanning Microscopy and Image Analysis

In whole LNCaP and PC3 cells, analysis of the spatial relationship between the proteins under study and the total DNA was performed by dual-color immunofluorescence staining as previously reported [15]. Proteins were labeled using a primary antibody against either special AT-rich sequence-binding protein-1 (SATB1) (1∶20 dilution, Santa Cruz) or poly (ADP-ribose) polymerase (PARP) (1∶50, Santa Cruz) with CF^TM^555 goat anti-rabbit IgG (1∶200 dilution, Biotium) as the secondary antibody, while DNA was labeled with 5 µM SYTOX Orange (Molecular Probes). In order to study the interactions between the NM proteins and MAR sequences, we performed immuno-FISH on NM extracted directly onto slides following the procedure described above. This immuno-FISH technique involved simultaneous staining of the NM proteins and DNA sequences and consists of two parts. In the first part (FISH), the MAR sequences were visualized using an XmnI biotinylated probe as reported above (see section halo-FISH). In the second part, the NM proteins were detected by immunofluorescence using rabbit anti-SATB1 (1∶15 dilution, Santa Cruz), rabbit anti-PARP (1∶40, Santa Cruz) or goat anti-lamin B (1∶20 dilution, Santa Cruz). Immunolocalization of the protein-MAR complexes was carried out using a cocktail of labeled secondary antibodies (CF^TM^633 anti-rabbit Ig, 1∶100 dilution and Alexa 488 anti-goat Ig, 1∶100 dilution) and streptavidin conjugated with CF^TM^555 (1∶100 dilution). All confocal images were collected according to the Nyquist criterion as 3D data sets (z-stacks) with a step size of 250 nm using an Olympus FV-500 laser scanning confocal microscope equipped with a 60×/1.4 NA oil-immersion objective. Image processing and co-localization analysis were performed as previously described [15] using WCIF ImageJ v1.43 software that provides both the Pearson’s correlation coefficient (R) and the co-occurrence Manders’ coefficients (M1 and M2).

**Figure 5:**
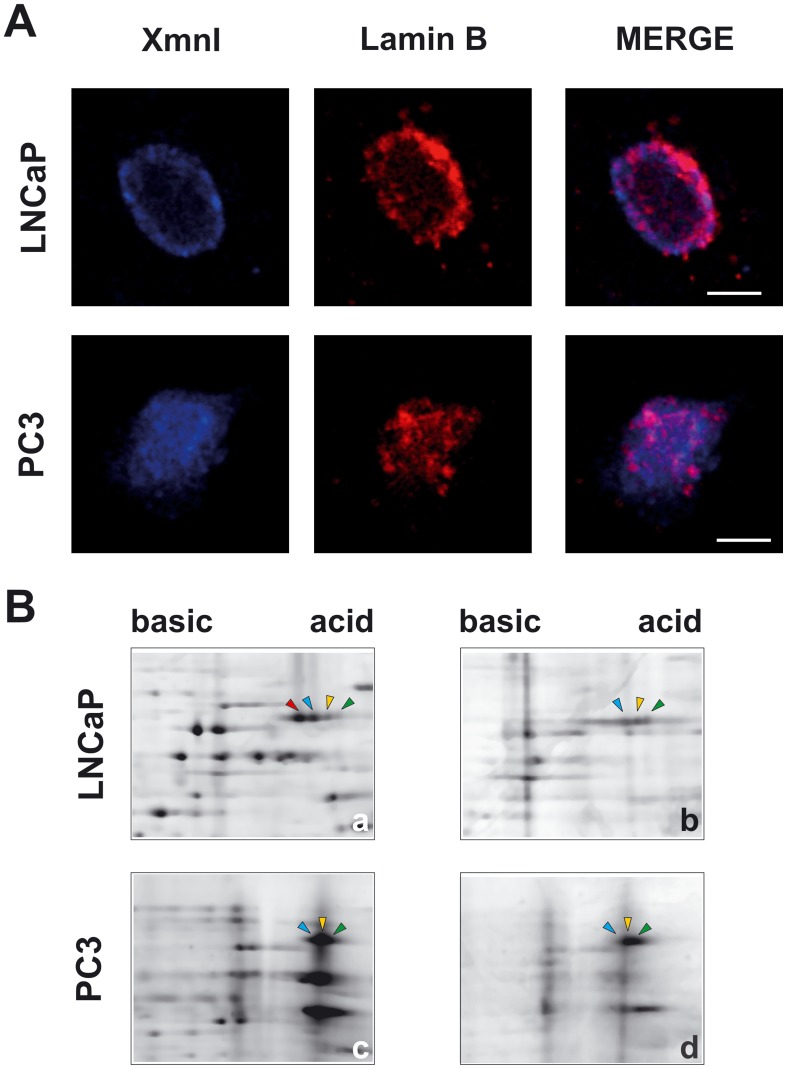
Spatial distribution and phosphorylation level of lamin B in the NM. (A) Representative confocal microscope images of lamin B (red) and XmnI sequence (blue) in the NM extracted *in situ* and stained by immuno-FISH. The bars correspond to 5 µm. (B) Magnified section of 2D-PAGE stained with SYPRO Ruby (a, c) or Pro-Q Diamond that selectively stains only phosphoproteins (b, d). The arrowheads indicate the various isoforms of lamin B. The same color corresponds to the same isoform in two cell lines. In PC3 cells, the non-phosphorylated peptide present in LNCaP cells disappeared (red arrowheads).

## Results

### The Expression of NM Proteins Binding MARs Depends on Differentiated State of PCa Cell Line

The NM proteins extracted from LNCaP, 22Rv1 and PC3 cells were separated by 1D-PAGE and a screen of binding to the XmnI sequence was carried out. In all cell lines examined, three groups of proteins (centered around 107, 71 and 55 kDa, respectively) that strongly bound DNA were identified (Fig. 1A, arrows). No qualitative changes were appreciable among the three cell lines; nevertheless, the quantity of DNA binding the NM proteins was significantly higher in the least aggressive cells (Fig. 1B).

**Figure 6:**
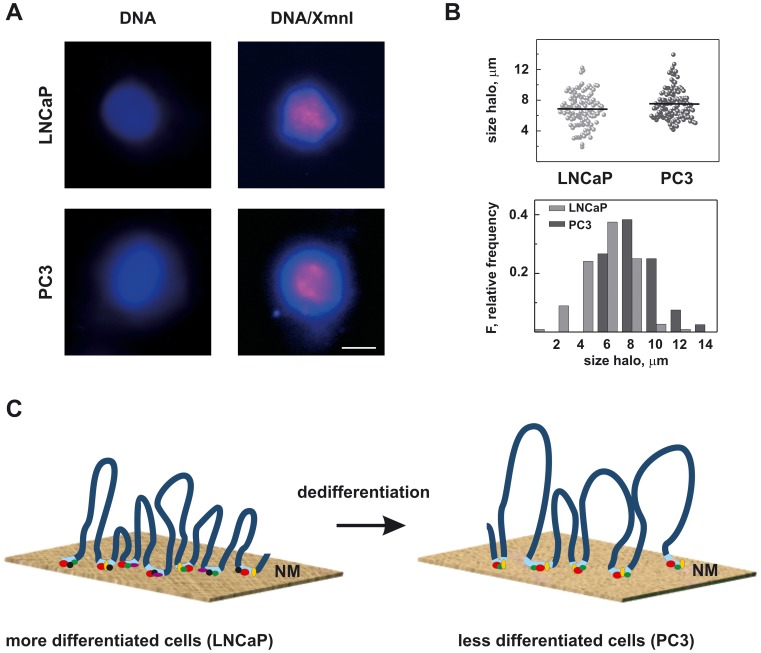
DNA loop organization in LNCaP and PC3 cells. (A) Representative nucleoids stained either by DAPI (blue) to visualize only total DNA (left panels) or by halo-FISH to highlight the XmnI sequence (red) and counterstained with DAPI to detect total DNA (blue).The bar corresponds to 10 µm. (B) Scatter plot showing the distribution of DNA halo size. Horizontal lines indicate the mean values obtained measuring for each cell line at least 100 nucleoids. The average DNA halo size ± SE was 6.8±0.2 for LNCaP cells and 7.5±0.2 for PC3 cells, respectively (P = 0.009). The bottom panel shows the frequency distribution of the halo radii grouped in intervals of 2 µm. (C) A schematic model of the interrelationship between the loops and the NM in the dedifferentiation of PCa cells. In more-differentiated cells (LNCaP) the NM is well organized with several proteins bound to MAR sequences. In PC3, where some structural regularities of the NM disappear, a smaller number of protein species bound the MARs and so a larger DNA loop is anchored to the NM.

polypeptides, we further characterized the expression of the NM proteins by 2D-PAGE followed by 2D SWB analysis. The overall expression profiles obtained from the 2D gels were rather similar; however, an analysis in depth showed that the complexity of protein pattern was inverse to grade of cell differentiation. PC3 cells showed the more complex NM protein pattern and 22Rv1 displayed an intermediate pattern between LNCaP and PC3 cells (Fig. 1C, E and G). On average, 725 protein spots were detected in the NM isolated from LNCaP cells (range 598–781), 847 (range 808–909) from 22Rv1 cells and 918 (range 727–1142) from PC3 cells. The Venn diagram reported in Fig. 2A shows that the majority of the spots visualized in 2D-PAGE match in three cell lines, in agreement with the result, already reported, that more than 70% of the NM proteins are common between cell lines and PCa tissues; these proteins are the component of NM cell-type specific [9], [16]. The spots differently expressed in 22Rv1 and PC3, with respect to LNCaP cells, were 98 and 155, respectively. In particular, 22 spots were over-expressed and 76 were under-expressed in 22Rv1 cells and 98 were over-expressed and 57 were under-expressed in PC3. This observation confirms previous results indicating that the NM protein pattern undergoes changes with cell differentiation [16] and increases in complexity in the passage from more- to less-differentiated tumors [7], [8], [17].

When 2D gels were analyzed by SWB, several NM proteins strongly bound the XmnI sequence (Fig. 1D, F and H). The bond was a sequence-specific rather than an aspecific (electrostatic) interaction because after incubation of the membranes overnight in 2 M NaCl the binding was still detectable (data not shown). In accordance with the 1D analysis, a global decrease in DNA binding was observed in function of cell differentiation: about 136 MAR-binding spots were visualized in LNCaP, 79 in 22Rv1 and 63 in PC3 cells. As a whole, this analysis shows that an increase in the complexity of the NM protein pattern is synchronous with a decrease of the number of proteins binding the XmnI sequence. Indeed, these latter were 19, 9 and 7% of the total NM protein expressed for LNCaP, 22Rv1 and PC3 cells, respectively (Fig. 2 B–D).

The principal XmnI binding spots were identified by WB and so it was possible to assign an identity to the proteins detected by 1D SWB. The first group, at higher molecular weight, corresponds to the co-migration of PARP1, heterogeneous nuclear ribonucleoprotein (hnRNP) U, MARPa and Matrin3; the second group corresponds to MARPb, lamin A, lamin B and hnRNP M; and the third group corresponds to PARP2, hnRNP I, hnRNP K and the fragments of PARP1 and SATB1.

The comparison among the 2D SWBs of the different cell lines understudy show that in addition to an alteration of number of proteins expressed also the signal intensity of DNA binding of some of their change. The more evident changes were between LNCaP and PC3 cells whereas, also in this case, the behavior of 22Rv1 cells was intermediate. Therefore, the following analyses were carried out comparing the two cellular more dissimilar i.e. LNCaP vs. PC3.

### The Expression and Localization of PARP and SATB1 Depend on the Level of Differentiation of PCa Cells

In PC3 cells with respect LNCaP, the signal intensity of DNA binding to Matrin3, SATB1 fragment, hnRNP U and all basic hnRNPs decreased. This decrease could be due to down-expression of the basic hnRNPs (which were among the 57 spots found to be under-expressed in PC3 cells), a change in the ratio of the expression of different isoforms (for example, for hnRNP U and Matrin3) or a different pattern of fragmentation (for SATB1). Vice versa, the expression of PARP2, MARPa and MARPb increased. Although these latter two proteins were present in such a small amount that they often were not detected by silver staining (Fig. 1C and G), they bound very strongly to the MARs. Unfortunately, as already reported [4], MARPa and MARPb haven’t been indentified yet and they are very likely to be novel uncharacterized proteins. So, we have quantified the expression of the proteins that differentially bound the XmnI sequence by 1D WB analysis using commercially available antibodies.

Diagrams illustrating the relative amounts of proteins and representative WB experiments are shown in Fig. 3. For hnRNP U and Matrin3, only a trend towards a decrease can be observed in PC3 with respect to LNCaP cells; vice versa, both PARP and SATB1 undergo significant modifications in their expression. Higher PARP expression was detected in PC3 cells, and the protein was in its native state with only a small quantity (about 19%) of the characteristic breakdown to 89 kDa [18] detected. Higher expression of this protein in PC3 cells (which represent a more undifferentiated phenotype with respect to LNCaP cells) is in agreement with the inverse correlation between the degree of cell differentiation and PARP activity [18].

In regards to SATB1, two prominent bands of nearly equal intensity at 89 (native SATB1) and 54 kDa are detectable in LNCaP cells. In PC3 cells, the expression of the full length protein decreases and three fragments at 62, 54 and 43 kDa are present. Additionally, the intensity of these three bands decreases to 60% with respect to intact SATB1, indicating that not only was the protein differently expressed between the two cell lines, but that both the intensity and the pattern of fragmentation were different as well (Fig. 3). The most common SATB1 degradation products are two fragments of about 70 and 30 kDa [19], even though other fragments, with molecular weights very close to those observed in our experiments, are also reported [20], [21]. The polyclonal goat antibody used in this study recognizes the SATB1 N-terminus; therefore, we expected to detect the bands corresponding to full-length SATB1 and the shorter peptides. Instead, in LNCaP cells the principal degradation product was a 54 kDa fragment that could correspond to the C-terminal deletion of amino acid 494 (Mr calculated 54,915) that contains the domains needed for localization at the NM and for binding to the MARs [22]. As a confirmation of what above said, in 2D SWB (Fig. 1D), we detected a SATB1 fragment binding the XmnI sequence at approximately 54 kDa. Moreover, the existence of a different protein fragmentation pattern in PC3 with respect to LNCaP is not due to the presence of apoptotic populations because we did not detect any DNA fragmentation nor apoptotic bodies in cell preparations (data not shown). It is therefore possible that the cleavage patterns are highly dependent on the particular differentiative and proliferative state of a cell [21], [23]. The reason why the whole SATB1 does not migrate on 2D despite migrating in 1D WB experiments is presently unknown. It could depend on the low concentration of the protein in the solution used to load samples into gel strips for the isoelectric focusing, even if solubility problems cannot be excluded.

To determine whether the distribution and interaction of PARP and SATB1 with the DNA and/or MAR sequences change as a function of the degree of cell differentiation, we performed confocal microscopy analysis.

In whole LNCaP and PC3 cells, PARP (visualized in green in Fig. 4A) was present in the nucleoplasm where it displayed a homogeneous granular staining, in agreement with previous investigations [24]. Merged images showed that PARP co-localized with DNA in both cell lines (R = 0.93 and 0.92 for LNCaP and PC3, respectively). When we performed confocal analysis of the NMs prepared *in situ*, marked differences in PARP distribution patterns were observed (Fig. 4B). In LNCaP cells, PARP was mainly distributed in punctuate sites, whereas in PC3 cells, where higher signal intensity was always detectable, the protein was more homogenously distributed. The XmnI sequence also localized differently in the isolated NMs. In LNCaP cells, the immuno-FISH analysis showed that the XmnI sequence is organized in speckles concentrated in the peripheral zone of the NM forming a well-defined ribbon. By contrast, in PC3 cells the speckles were scattered throughout the whole NM. Densitometric analysis of the fluorescence signals along the line scans showed an evident overlap of the blue (XmnI) and green (PARP) channels. Moreover, the parameters reported in Fig. 4C (panel b) indicated that in NM prepared from PC3 cells, where PARP is expressed at higher levels, R and M2 significantly increased, thus indicating a major colocalization between PARP and the XmnI sequence in PC3 cells with respect to LNCaP cells.

In the whole cells, SATB1 is present in the nucleoplasm in both PC3 and LNCaP cells, where it showed a speckled staining pattern as previously observed [25]. Only minimal fluorescence intensity was visible in the cytoplasm (Fig. 4D). SATB1 colocalization with DNA was very high and significantly increased in PC3 cells with respect to LNCaP cells (R = 0.91 and 0.89, respectively). The cage-like network surrounding the heterochromatin that is typical in thymocytes [26] was not visible, as already reported for embryonic stem cells by Savarese *et al*. [25]. In the NM (Fig. 4E), the distribution of SATB1 was maintained. In NM prepared from LNCaP cells, the colocalization between SATB1 and the XmnI sequence was poor (R = 0.38) and only present in a few foci at the NM periphery. In PC3 cells, the colocalization between the SATB1 and the MAR sequence was significantly higher (R = 0.54), and M2 (the fraction of XmnI overlapping with SATB1) increased from 0.66 to 0.90, indicating that the majority of MARs colocalize with SATB1 (Fig. 4F, panel b).

### The Phosphorylation Level of Lamin B in PCa Cells Depends on the Grade of Differentiation

Lamin B binds MAR sequences [27] and has an important role in chromatin compartmentalization; moreover, we have shown that in human PCa, lamin B undergoes a significant increase correlated to the Gleason score [28]. For these reasons, we observed the fate of lamin B during prostate cell differentiation. The expression of lamin B, determined by quantitative 1D WB, was not affected in the two cell lines; moreover, the protein was always in a native form and no pattern of fragmentation was detected (data not shown). Confocal analyses, reported in Fig. 5A, revealed that in the LNCaP cells lamin B showed an intense fluorescence confined to the NM periphery to form a dotted rim, while only few and of minor spots were visible in the internal NM. In PC3 cells, the fluorescence pattern consisted of dots scattered throughout the entire NM and a significant decrease from 0.64±0.01 (mean value ± SE) to 0.58±0.02 (P = 0.018) in the fraction of XmnI overlapping lamin B was detected. It is known that the subnuclear distribution of lamin B mainly depends on its phosphorylation state [29]; therefore, we carried out a characterization of the grade of lamin B phosphorylation by 2D-PAGE. We compared the Sypro Ruby dye signal intensity with the Pro-Q Diamond dye signal (which selectively stains phosphoproteins) for each lamin B spot. A representative electrophoretic pattern obtained for LNCaP and PC3 is reported in Fig. 5B. In LNCaP, four different isoforms and three phosphopeptides were present, and the non-phosphorylated peptide was the most prominent isoform (about 59% of the total lamin B expressed). In PC3 cells the non-phosphorylated peptide was not expressed, directly showing the occurrence of an increase in lamin B phosphorylation.

### The LNCaP Halo of DNA Loops Differs from that of the PC3 Cell Line

Attachment of MARs onto the NM is responsible for the loop domain structure of chromatin [3]; consequently, we hypothesized that the changes in the MAR-binding proteins reported above could modify the loop dimensions. To test our hypothesis, we took advantage of the FISH technique to assess the nuclear halo. Nuclei purified from two cell lines were extracted with 2 M NaCl to remove the histones, thus allowing us to obtain nucleoids. Under these conditions, the DNA forms a halo extruding from the nucleus made up of loops attached to NMs at their bases. Total DNA was stained with DAPI, while the XmnI sequence was visualized by FISH.

No difference in the distribution of the XmnI sequence was observed, being in this case also in presence of all the cellular DNA and not only the one bound to the NM. Surprisingly though, a large difference in the halo radius was clearly evident (Fig. 6A). Measuring the dimensions of more than 100 nucleoids per cell line, we found that in LNCaP cells the average halo size was significantly smaller than in PC3 cells (Fig. 6B). Since the average DNA halo size in the first approximation can be correlated with the average loop size [30], and assuming that the grade of supercoiling of the loop is equal in both cell lines, we can infer that in PC3 cells the DNA loops are larger. This result shows that chromatin loop organization is different depending on cell type, as has already been reported by de Belle *et al*. [12], and that the presence of larger loops may correlate with the lower number of NM proteins binding MAR sequences as reported in Fig. 1.

## Discussion

In the present analysis, we provide evidence that shows, for the first time, that during PCa cell differentiation the binding between the NM proteins and MAR sequences is dynamic and inversely correlated with the cellular differentiation. In less differentiated and more aggressive cells (PC3), a smaller number of proteins bind the MARs and the average value of dimension of the loop increase. These modifications are synchronous with a global increase in the number and level of protein expressed in the NM indicating a higher transcriptional activity. These findings are consistent with the association between induction of gene expression and large-scale chromatin unfolding that has been shown in mammalian cells [31]. It is well known that loss of differentiation is accompanied by the remodeling of nuclear organization: the chromatin decondenses and the heterochromatin domains translocate from the periphery towards the internal nuclear regions [32]. In addition, the complexity of the NM protein pattern increases [7], [8], [17], and alterations in the NM ultrastructure are detectable [5], [16]. Moreover, Maya-Mendoza *et al*. [33] found that in natural aging of the rat liver, the average DNA loop size gradually decreases, and DNA loops increase in number with the gradual loss of proliferating potential and with the progression towards terminal differentiation. More recently, it has been shown that in breast cancer cell lines the dimension of chromatin loops are larger as compared to that of normal cells [34]. These results are in agreement with the data reported above.

In a model for the interrelationship between the MARs and DNA loop anchorage to the NM, it has been hypothesized by Razin’s group that the size of the chromatin loops increases with differentiation and decreases with progression to malignancy [35]. This is in apparent contrast with the increase in the dimension of the loops that we found in less-differentiated PC3 cells; however, as the same authors suggest, the loop organization could show cell-type specificity, which could be present during prostate tumor progression. Moreover, the halo dimensions, from which we have inferred the loop dimensions, could be strongly influenced by both the presence of giant loops, namely clusters of highly expressed genes [36] and the fraction of DNA embedded in the NM [37]. In LNCaP cells, where a major number of NM proteins bind MAR sequences, a larger fraction of DNA could be embedded in the NM resulting in a shorter average DNA-loop.

In the differentiation model of prostate tumor progression used in this study, the changes in nuclear organization could be triggered by coordinated modifications in the expression of a few proteins (i.e. PARP, SATB1) and in the increased phosphorylation of lamin B. These modifications altering the NM structure could change the interactions between the NM and the MARs, consequently determining variations in the loop dimensions and therefore in gene expression, which may ultimately give rise to a more aggressive phenotype. It is important to underline that although the proteomic approach has been able to reveal several hundred NM proteins and that the expression of many of these changed during tumor progression, only a very restricted number of MAR-binding proteins, belonging to facultative MAR-binding proteins (i.e. cell type- and activity-related or cell differentiation depending [2]), seem to play key roles in this process.

The behavior of PARP in PCa differentiation presented in the current study is in agreement with the results of Galande and Kohwi-Shigematsu that detected a strong BUR-specific binding activity of PARP in malignant, poorly differentiated breast carcinoma compared to well-differentiated tumor samples [38]. Furthermore, transcriptional down-regulation of androgen receptor in the aging rat liver and in oxidatively stressed hepatoma cells is regulated by PARP1 in association with hnRNP K [39], which is the NM protein that has a pivotal role in PCa and the one that we found to be highly expressed in less-differentiated tumors [9].

SATB1 is the best characterized MAR-binding protein, and its roles in the higher order of chromatin loop organization and global transcriptional regulation have been widely documented [40]; however, data on its expression level and its role in tumor development are still conflicting. Han *et al*. [41] showed that SATB1 expression levels correlate with poor prognosis in breast cancer and promote tumor growth and metastasis. In contrast, a more recent study by Iorns *et al*. [42] found that SATB1 was not associated with breast cancer pathogenesis, and that significant loss of SATB1 expression was found in squamous preinvasive lesions and in non-small cell lung cancers [43]. Our results demonstrate that in less-differentiated PCa cells, a decrease in the expression of SATB1 in the NM and a minor fragmentation are associated with higher colocalization with the XmnI sequence, thus supporting the model that SATB1 acts as a structural platform that provides a base for chromatin loops [36] and suggesting that, in PCa cell differentiation, the level of interaction of SATB1 with MAR sequences could be more important than its expression level.

Apparent invariance in the level of expression of lamin B together with an increase in phosphorylation are in agreement with our previous hypothesis that the lamin scaffold in the nucleus might represent a building block of a permanent scaffold structure, whereas post-translational modification of lamins could be correlated with the levels of differentiation and proliferation [5], [10].

In conclusion, our data provide evidence that the interactions between the NM and MAR sequences are involved in PCa differentiation and can play important roles in the androgen-independent phenotype; work is in progress in our laboratory to extend these findings to prostate cancer tissues with different Gleason scores. Our hypothesis is in line with recent results demonstrating that translocation breakpoints in PCa contain androgen receptor binding sites [44], being known that break cluster regions often map to MARs. The proteins bound to MAR sequences that are involved in these processes could be important tools in understanding the PCa carcinogenesis process and could be novel targets for androgen-independent PCa therapy.
